# Precision weighting of cortical unsigned prediction error signals benefits learning, is mediated by dopamine, and is impaired in psychosis

**DOI:** 10.1038/s41380-020-0803-8

**Published:** 2020-06-24

**Authors:** J. Haarsma, P. C. Fletcher, J. D. Griffin, H. J. Taverne, H. Ziauddeen, T. J. Spencer, C. Miller, T. Katthagen, I. Goodyer, K. M. J. Diederen, G. K. Murray

**Affiliations:** 1 Department of Psychiatry, University of Cambridge, Cambridge, UK; 2 Wellcome Trust MRC Institute of Metabolic Science, Cambridge Biomedical Campus, Cambridge, UK; 3 Cambridgeshire and Peterborough NHS Trust, Cambridge, UK; 4 Department of Psychosis studies, Institute of Psychiatry, Psychology and Neuroscience, King’s College London, London, UK; 5 Department of Psychiatry and Psychotherapy, Charité—Universitätsmedizin Berlin, Corporate member of Freie Universität Berlin, Humboldt-Universität zu Berlin, Berlin Institute of Health, Berlin, Germany

**Keywords:** Neuroscience, Schizophrenia, Psychology

## Abstract

Recent theories of cortical function construe the brain as performing hierarchical Bayesian inference. According to these theories, the precision of prediction errors plays a key role in learning and decision-making, is controlled by dopamine and contributes to the pathogenesis of psychosis. To test these hypotheses, we studied learning with variable outcome-precision in healthy individuals after dopaminergic modulation with a placebo, a dopamine receptor agonist bromocriptine or a dopamine receptor antagonist sulpiride (dopamine study *n* = 59) and in patients with early psychosis (psychosis study *n* = 74: 20 participants with first-episode psychosis, 30 healthy controls and 24 participants with at-risk mental state attenuated psychotic symptoms). Behavioural computational modelling indicated that precision weighting of prediction errors benefits learning in health and is impaired in psychosis. FMRI revealed coding of unsigned prediction errors, which signal surprise, relative to their precision in superior frontal cortex (replicated across studies, combined *n* = 133), which was perturbed by dopaminergic modulation, impaired in psychosis and associated with task performance and schizotypy (schizotypy correlation in 86 healthy volunteers). In contrast to our previous work, we did not observe significant precision-weighting of signed prediction errors, which signal valence, in the midbrain and ventral striatum in the healthy controls (or patients) in the psychosis study. We conclude that healthy people, but not patients with first-episode psychosis, take into account the precision of the environment when updating beliefs. Precision weighting of cortical prediction error signals is a key mechanism through which dopamine modulates inference and contributes to the pathogenesis of psychosis.

## Introduction

A common theme in contemporary theories of brain function, ranging from perception [1] to reinforcement learning [2], is an emphasis on the critical role in inference played by predictions based on prior knowledge [1–8]. According to these theories, predictions and incoming sensory input each have an associated precision (inverse variance) reflecting their confidence or reliability. Predictions and sensory input are thought to be compared against one other, generating a discrepancy signal termed the prediction error, which indicates the difference between the expectation and sensory input. Such prediction error signals update prior beliefs in a manner that is weighted by their associated precision, such that more is learned from precise and reliable prediction errors compared with noisy and unreliable prediction errors [2, 4, 6]. Several theorists have suggested that neuromodulator systems, including dopamine, play an important role in mediating the precision of these prediction errors, and that impaired precision-weighting of prediction errors (through dopaminergic or other neuromodulator dysfunction) may be part of the cascade that results in psychotic symptoms [3, 4, 6, 9]. However, to the best of our knowledge no direct evidence for this hypothesis exists. Specifically, whilst several neuroimaging studies have indicated abnormal brain prediction error signals in schizophrenia and related psychoses [10–13], none of these studies have addressed precision weighting of prediction errors in patients.

In the context of reinforcement learning models, a distinction can be made between two types of prediction errors. First, the signed prediction error indicates whether an outcome is better or worse than expected, and thereby plays a crucial role in changing the value allocated to cues, thereby guiding future decisions [14–21]. A second type of prediction error, the unsigned prediction error, signals the degree of surprise without indicating valence (better/worse than expected). In addition to signed prediction errors, unsigned prediction errors are included in various reinforcement learning models to control how much should be learned from new information. Large unsigned prediction errors signal that the brain’s model of the world is inaccurate, thereby increasing the amount that is learned from new information. This can be achieved in various ways, including a non-Bayesian approach by using a dynamic learning rate parameter [22, 23] or a Bayesian approach by decreasing the precision of prior beliefs [24, 25] across different levels in the hierarchy so that new sensory information has more of an impact on learning [2]. In these hierarchical models both signed and unsigned prediction errors are weighted by their precision. Whilst evidence has been provided for a dopamine-mediated precision-weighted signed prediction error in learning [15, 16, 26], no such evidence exists for dopaminergic modulation of the precision weighting of unsigned prediction errors. This is despite many computational theorists hypothesising both a role for neuromodulator systems in precision weighting of unsigned prediction errors [3, 6], and dysfunctional precision-weighting as a key contributor to the pathogenesis of psychosis [3, 9, 27, 28].

Here we studied whether and how precision weighting of signed and unsigned prediction error signals is disrupted in psychosis. As the presence of precision weighting of unsigned prediction error signals has previously been largely speculative, we first tested whether unsigned prediction errors are indeed coded relative to their associated precision in the cortex of healthy individuals and whether dopamine modulates the precision of these prediction error signals. Our methods elicit reliable measures of prediction error, as we use a task where prediction error is directly observable, rather than inferred as a latent variable as is common in many paradigms. To probe the role of precision-weighted prediction errors in learning, and the influence of dopamine, we employed pharmacological modulation in healthy volunteers, combined with fMRI, associative learning and computational modelling. We next examined how individual differences in computational learning signals and brain precision-weighting signals relate to clinical psychosis, and psychotic-like thinking in health (schizotypy).

## Methods

### Participants and intervention—dopamine study

Fifty-nine healthy volunteers completed the pharmacological fMRI study (Table 1, see also ref. [16] and Supplement); all provided written informed consent. Prior to scanning, participants received a single dose of the D2-antagonist sulpiride (600 mg), the dopamine agonist Bromocriptine (2.5 mg) or placebo, in a double-blind fashion. The study received approval from the Cambridge South NHS Research Ethics Committee (12/EE/0039).

**Table 1:** Demographics for dopamine study.

	Dopamine study						
	Placebo		Sulpiride (antagonist)		Bromocriptine (agonist)		*p* value
*N*	20		20		19		
Male	9 (11)		12 (8)		10 (9)		
	Mean	SD	Mean	SD	Mean	SD	
Age	23.9	4.8	24.8	4.5	23.7	4.3	
Reverse digit span	6.2	1.5	5.7	1.5	6.8	3.8	*p* = 0.40
Schizotypy	16.6	10.1	15.4	11.5	15.6	11.2	*p* = 0.99

### Participants—psychosis study

Healthy volunteers (HCS, *n* = 30, average 22.6 years, 15 female) without a history of psychiatric illness or brain injury were recruited as control subjects. Healthy volunteers did not report any personal or family history of neurological, psychiatric or medical disorders. As in our previous work [10], we recruited participants with first-episode psychosis (FEP) with active delusions or hallucinations (PANSS P1 or P3 > 2) (FEP, *n* = 20 average 24.8 years, six female) or participants at-risk of psychosis (at-risk mental states (ARMS), *n* = 24, average 21.5 years, eight female) who were recruited from the Cambridgeshire early intervention in psychosis service (Table 2). In addition, potential at-risk participants were identified on the basis of belonging to a help-seeking, low-mood, high schizotypy sub-group from the Neuroscience in Psychiatry Network cohort [29] or through advertisement via posters displayed at the Cambridge University counselling services. Individuals at-risk for psychosis met ARMS criteria on the CAARMS interview in the past 6 months [30]. Sample size was determined based on our prior studies [10, 11]. All participants gave written informed consent. The study received NHS research ethics approval (West of Scotland REC 3, IRAS 137762).

**Table 2:** Demographics for psychosis study.

Group	Healthy controls (HCS)		At-risk mental state (ARMS)		FEP (First-episode psychosis)		
*N*	30		24		20		
	Mean	SD	Mean	SD	Mean	SD	*p* value
Age	22.6	3.5	21.0	3.5	24.9	5.2	*p* = 0.009
Male	16		18		17		*p* = 0.044
IQ	118.8	10.5	118.7	11.7	106.3	20.2	*p* = 0.006
PANSS positive	7.2	0.8	14.0	3.00	20.6	5.4	*p* < 0.001
PANSS negative	7.2	0.9	13.5	6.2	15.5	8.4	*p* < 0.001
Taking antipsychotic medication	0/30		4/24		12/20		*p* < 0.001
Antipsychotic medication dosage (chlorpromazine equivalent dose)	0	0	55.5	64.3	353.5	184.54	*p* = 0.008

### fMRI task design

**Fig. 1:**
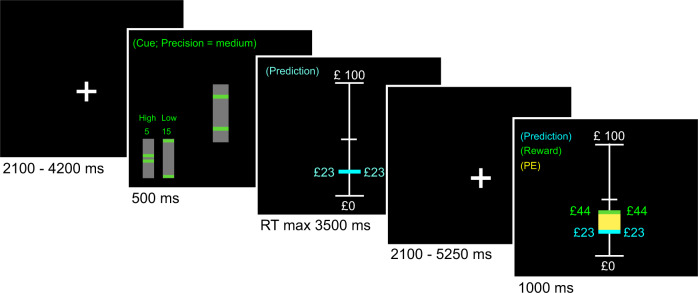
Example of a trial. The participants were instructed to learn the mean of a reward distribution. First a fixation cross was presented after which the participants were informed about the standard deviation (which indicated the precision) of the reward distribution. Subsequently the participants were asked to make a prediction regarding the upcoming reward, which was presented to the participant in combination with the prediction error (in yellow) after an anticipation period.

The task (see Fig. 1 and Supplementary methods) consisted of three sessions of 10 min each. Rewards were drawn from six different pseudo-Gaussian distributions that differed with respect to their precision (i.e., inverse variance) and expected value (i.e., mean of the distribution). For the dopamine study, the standard deviations from the distributions were 5, 10 and 15, corresponding precisions of 0.04, 0.01 and 0.004. For the psychosis study, the standard deviation of the distributions were either 5 or 15, corresponding to precisions of 0.04 and 0.004. Distributions were counterbalanced to ensure that the two conditions within each session differed with respect to the mean of the distribution and precision. Conditions were presented in short blocks, each including 4–6 trials. Each distribution consisted of 31 trials, resulting in 62 trials per session. Participants encountered two distributions per session (run) and were informed beforehand that each distribution had a different level of precision (low, medium or high precision, corresponding to precisions of 0.004, 0.01 and 0.04, although these exact numerical values were not revealed to participants). Furthermore, participants were instructed that the two distributions within a session would have different means. In the psychosis study there was no medium precision condition. Participants were presented with a cue that indicated the precision (high, medium or low) of the reward distribution used in the upcoming block. Participants were then required to predict the magnitude of the upcoming reward and received feedback after a delay. Optimal performance thus required the participant to estimate the mean of the distribution from which the rewards were drawn. For MRI acquisition details see Supplementary methods.

### Behavioural analysis and computational modelling

The mean performance error—the absolute value of (actual mean − predicted mean)—was our index of performance, which we compared across groups. We also fitted several reinforcement learning models to participants’ prediction sequences (see Supplementary methods). In brief, each model used a common updating rule in which predictions on a given trial depended on the prediction error and the learning rate on the previous trial. We implemented a Rescorla–Wagner (RW) reinforcement learning model with a fixed learning rate [31] and a Pearce–Hall (PH) model with a trial-wise, dynamic learning rate, which prescribes higher weighting of prediction errors (i.e., more learning) at the start of a task session compared with later trials [22]. In uncertain environments, it is optimal to decrease the weighting of prediction errors as learning progresses (once participants become more certain of their predictions) as prediction errors will continue to occur as a result of the imposed uncertainty. We additionally explored whether scaling prediction error to the reliability of the environment (i.e., precision weighting) benefitted learning by comparing models that scaled the prediction error term and models that did not. This results in six models: (1) simple RW, (2) RW with a scaled prediction error term, (3) PH model (decaying learning rate), (4) PH model with scaled prediction error term, (5) PH model with individual estimates of scaling term, (6) PH model with individual estimates of separate signed and unsigned prediction error scaling.

### Brain imaging analysis

We modelled the onsets of the cue and the outcome as events (i.e., delta functions of zero duration) and the onset of the prediction event (i.e., when participant could start making their prediction) as a single epoch lasting until they indicated their prediction. Each predictor was convolved with the standard canonical haemodynamic response function in SPM8. We used parametric modulation to identify neural correlates of unsigned prediction error responses by specifying the unsigned prediction errors for all outcome events. It is important to note that the prediction errors used in these analyses are simply the absolute difference between predicted reward and received reward. As such, the prediction errors did not depend on the behavioural modelling and therefore could not be influenced by any differences in the best-fitting model between groups. In a separate analysis, we also explored the coding of signed prediction errors in the psychosis study. The effect of dopaminergic drugs on the precision weighting of signed prediction errors has been published before [16]. Reward events were separately modelled for the different precision conditions to test for differences in precision weighting of prediction errors as evidenced by different sizes of slopes for the coding of unsigned prediction errors under different levels of certainty. Contrasts were created on the 1st level. As we were interested in the effect of precision but not mean reward, we collapsed all the different means for each precision condition, so there were two or three precision conditions in the psychosis study and dopamine study respectively, to be taken to the 2nd level (i.e., group level): see Supplement for details of group-level analysis.

## Results

### Study 1: dopamine modulation study

#### Environmental precision and dopamine D2 receptor antagonism modulate task performance

**Fig. 2:**
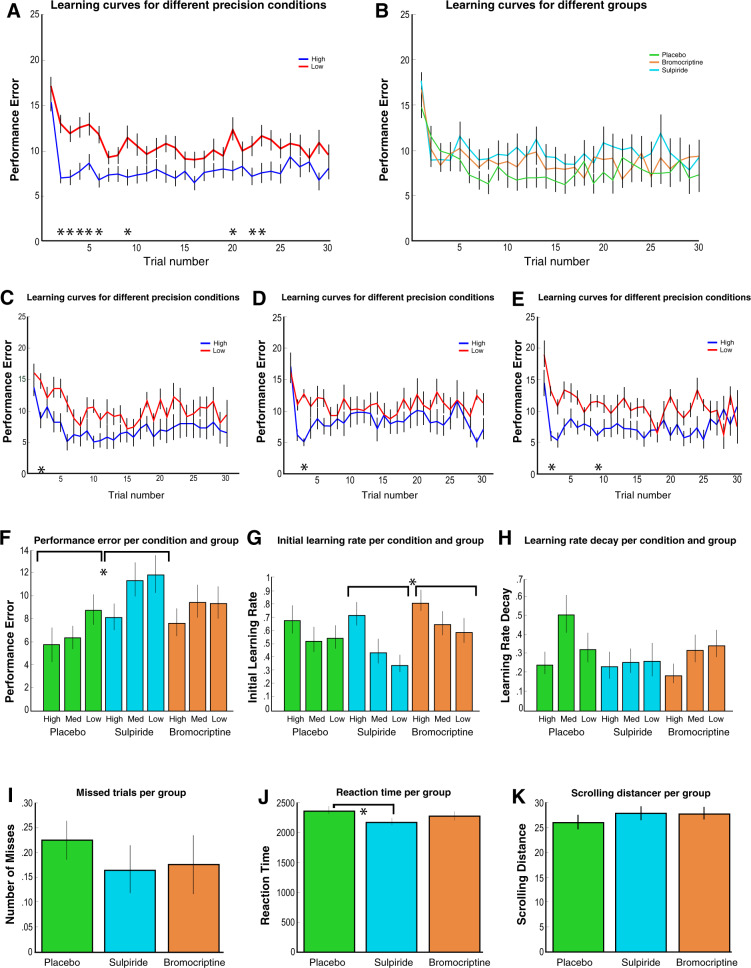
Behavioural results for the dopamine study. **a–e** display the average learning curves, reflecting the absolute distance between the actual mean of the distribution and participants’ estimate of the mean distribution over 30 trials averaged over the three sessions for each participant. The distance between the prediction and the actual mean of the distribution defines performance error. Therefore, lower values of performance error are better. Asterisks indicate Bonferroni-corrected significant differences across conditions. **a** Performance was significantly better in the high-precision condition compared with the low-precision condition when combing all participants, especially at the beginning of the experiment. **b** There were no clear differences between groups when analysing trial-by-trial performance. The placebo (**c**), sulpiride (**d**) and bromocriptine (**e**) group showed only a significant difference between the precision conditions one or two trials in the beginning of the experiment. **f** Averaging performance error across all trials, we see overall better performance in the placebo condition compared with the sulpiride condition, and higher performance in more precision conditions. **g** Initial learning rate parameters are displayed here for the Pearce–Hall model. Initial learning rates were higher in the Bromocriptine condition compared with the sulpiride condition. **h** Learning rate decay parameters from the Pearce–Hall model are displayed here. Precision and group did not affect the learning rate decay parameter. **i** No differences were found in number of missed trials, and **k** scrolling distance. **j** Reaction times were quicker in the sulpiride group. Error bars represent standard error of the mean.

Participants’ performance (the distance between the participant’s prediction and the mean of the distribution) improved when the precision of the reward distributions increased, with reduced average performance with sulpiride (Fig. 2a–f and Supplementary Fig. 1); trial-by-trial analysis revealed lower performance in low-precision conditions at the start of the experiment, but no significant group differences (Fig. 2a–e and Supplementary Fig. 1).

#### Reinforcement learning modelling of behavioural data indicates precision-weighted unsigned and signed prediction errors

Since formal learning models like the PH model suggest that unsigned prediction errors increase learning, we expect an interaction between unsigned and signed prediction error on participants’ trial to trial updates. The unsigned*signed prediction error interaction term was highly significant in predicting updates (*F*{1,10763} = 51.7, *p* < 0.0001), demonstrating the importance of unsigned prediction errors in learning (Supplementary Fig. 2). In all three medication groups a PH model with separately estimated precision-weighted signed and unsigned prediction errors best predicted behaviour (Supplementary Table 1). These results indicate that both unsigned and signed prediction errors are precision-weighted to facilitate efficient learning under uncertainty.

#### Unsigned prediction errors are coded in the superior frontal cortex (SFC) and pre SMA/dACC

**Fig. 3:**
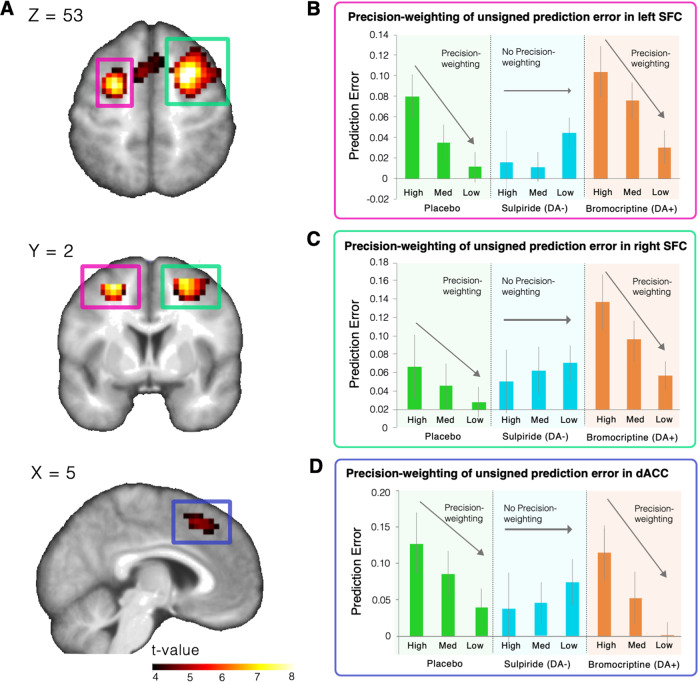
Brain imaging results for the dopamine modulation study. **a** Unsigned prediction errors were coded in bilateral superior frontal cortex and dorsal anterior cingulate cortex. The left side of the brain is the left side of the image. **b**–**d** When exploring these regions further, we find that unsigned prediction errors are coded in a precision-weighted fashion as indicated by the strong unsigned prediction error signal in the high-precision condition, which declines over the medium- and low-precision condition in the placebo and bromocriptine group. Importantly, sulpiride perturbed precision-weighting significantly in the left SFC. Error bars represent standard error of the mean.

We next explored where unsigned prediction errors are coded in the brain, in order to find the region of interest for our subsequent analyses focussing on the effect of dopamine on the precision weighting of prediction errors. Whilst correcting for whole-brain comparisons, unsigned prediction errors were coded in the frontal, parietal and occipital cortices (Fig. 3a and Supplementary Table 2). We used the left and right SFC and dACC clusters as ROIs to take forward for our analysis of dopaminergic effects on precision weighting. Secondary analyses examined these effects in occipital and parietal regions (Supplementary materials).

Precision weighting of unsigned prediction errors is mediated by dopamine in the SFC/dACC. To test whether precision and dopaminergic perturbations affected the coding of unsigned prediction errors, we extracted the parameter estimates (betas) of the unsigned prediction error parametric modulators from the left and right SFC and dACC cluster that showed a main effect of unsigned prediction error coding at whole-brain corrected *p*FWE < 0.01. We used a two-factor mixed model ANOVA with medication group as the between-subjects variable and precision condition as the within-subjects variable, using a linear contrast across precision conditions for the main effect of precision and interaction.

In the left SFC cluster, there was a significant interaction across precision conditions and medication group, suggesting that medication had a significant effect on precision weighting of unsigned prediction errors (*F*{2, 56} = 4.025, *p* = 0.023; Fig. 3b). There was a significant interaction between medication group (placebo vs. sulpiride) and precision condition (*F*{2, 37} = 5.44, *p* = .025), with less precision weighting in the sulpiride than in the placebo group, which suggests that sulpiride dampens precision weighting of unsigned prediction errors. Comparing the placebo and bromocriptine group, there was a significant effect of precision (*F*{1, 37} = 14.93, *p* < 0.001), but no significant effect of medication group (placebo vs. bromocriptine) (*F*{1, 37} = 2.781, *p* = 0.104) or interaction between medication group and precision (*F*{2, 36} = 0.02, *p* = 0.894). This finding suggests that left SFC unsigned prediction error signals are precision-weighted, but relatively unaffected by bromocriptine.

In the right SFC we did not find a significant interaction between medication and precision condition (*F*{2, 56} = 1.70, *p* = 0.193; Fig. 3c and Supplementary Fig. 3b). However, signal changes in the right SFC are largely the same as in the left SFC (see Fig. 3b). We did find a significant main effect of medication (*F*{2, 56} = 3.65, *p* = 0.032). This effect was driven by a stronger main effect of unsigned prediction error in the bromocriptine group compared with the placebo group (*F*{1, 37} = 6.740, *p* = 0.013), whereas the difference was only trend-level significant between the sulpiride and placebo group (*F*{1, 38} = 3.55, *p* = 0.067).

**Fig. 4:**
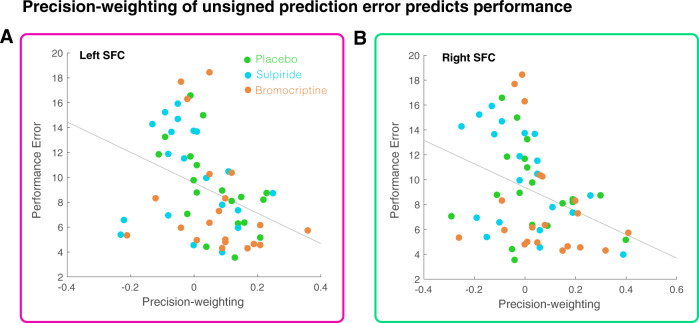
Cortical learning signals and performance. Precision weighting of unsigned prediction errors in the left SFC (**a**) and right SFC (**b**) correlates with performance (i.e., difference between mean of the reward distribution and predicted mean) on the task.

In the dACC we found a trend-level significant interaction between precision and medication (*F*{2, 56} = 2.81*, p* = 0.069; Fig. 3d and Supplementary Fig. 3c). Post hoc tests between the placebo and sulpiride group revealed a trend-level interaction between precision and medication (*F*{1, 38} = 3.043, *p* = 0.089). Testing the placebo and bromocriptine group revealed a significant effect of precision (*F*{1, 37} = 9.32, *p* = 0.004), but no effect of group (*F*{1, 37}=1.17, *p* = 0.29) or interaction (*F*{1,37} = 0.172, *p* = 0.68). A similar pattern was thus found in the dACC as in the left SFC (see Fig. 3b, d). The degree of cortical precision-weighting correlated with task performance (controlling for group), such that higher precision-weighting relates to better performance (Fig. 4, Left: Rho = −0.45, *p* < 0.001; Right: Rho = −0.40, *p* = 0.002; dACC: Rho = −0.25, *p* = 0.055). There were no whole-brain effects of group on precision weighting.

### Study 2: psychosis study

#### FEP is associated with decreased overall performance and less benefit from more precise information

**Fig. 5:**
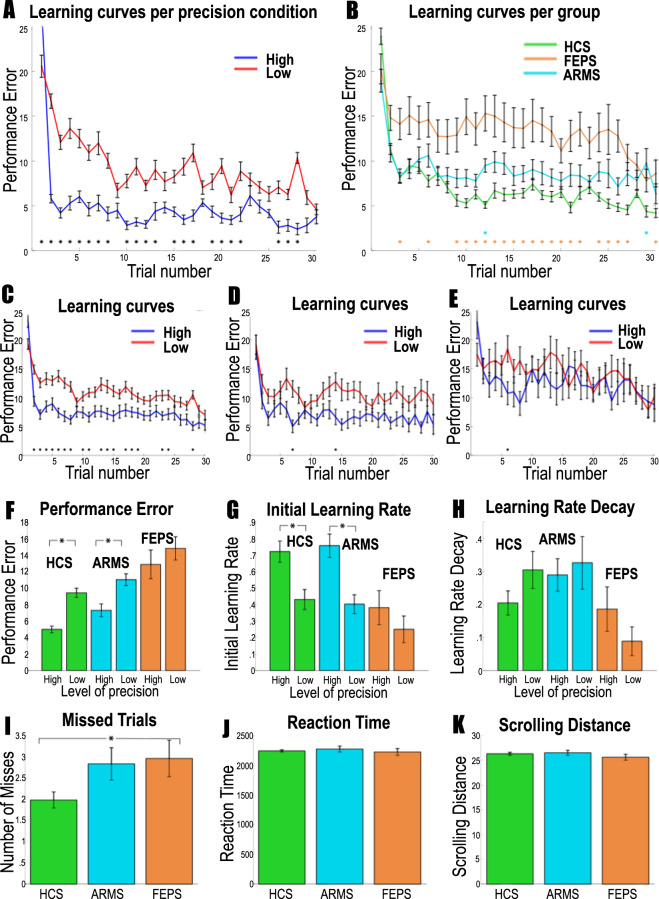
Behavioural results for the patient study; green bars are healthy controls, blue ARMS and orange first-episode psychosis. **a–e** display the average learning curves, reflecting the absolute distance between the actual mean of the distribution and participants’ estimate of the mean distribution over 30 trials averaged over the three sessions for each participant. The distance between the prediction and the actual mean of the distribution defines performance error. Therefore, lower values of performance are better. Asterisks indicate Bonferroni-corrected significant differences across conditions. **a** Performance was significantly better in the high-precision condition compared with the low-precision condition when combing all participants, especially at the beginning of the experiment. **b** HCS performed better than FEP, but not compared with ARMS. The colour of the asterisk indicates a significant difference of the patient group with HCS. Healthy controls (**c**) showed a significant difference between the precision conditions, whereas the patient groups did not (**d** ARMS, **e** FEP). **f** Averaging performance error across all trials, HCS and ARMS perform better than FEP, and benefitted from more precise information, whereas FEP did not. **g** Initial learning rates are displayed here for the Pearce–Hall model. Learning rates were higher in the high-precision condition compared with the low-precision condition in HCS and ARMS, but not for FEPS, although an interaction was not significant. **h** Learning rate decay parameters are displayed here for the Pearce–Hall model. Precision did not affect the learning rate decay parameter. **i** HCS had slightly fewer missed trials than FEP. **j** Reaction times were equal across groups. **k** Scrolling distance was equal across groups. Error bars represent standard error of the mean.

We explored the difference between participants’ estimates of the mean and the actual mean across trials, and tested for significant differences across groups and precision conditions while correcting for multiple comparisons using a Bonferonni correction. We found better performance when precision was high in healthy control participants and ARMS individuals but not in participants with FEP. We also found decreased performance in the FEP group compared with controls (see Fig. 5a–f and Supplementary Fig. 4). There was a trend-level difference in number of missed trials, suggesting that on an average the healthy controls missed one trial less than the other groups. There were no other significant differences in RT and scrolling distance (see Fig. 5i–k and Supplementary Fig. 4).

### FEP is associated with a lack of precision weighting as revealed by computational modelling

We found that for the HCS and ARMS participants the best model of behaviour was the PH model with a precision-weighting parameter for both the signed and unsigned prediction error term. However, for the FEP group a simple RW learning rule without precision weighting of prediction error was the best fit, suggesting that the FEP group specifically is not precision-weighting prediction errors (Supplementary Table 3a, b and Supplementary Figs. 5–7). Formal tests of differences in model fit revealed mostly non-significant differences between models for the FEP group (Supplementary results). The difference in degree of precision weighting between groups is further supported by the observation that HCS and ARMS participants show higher learning rates in the high-precision condition, whereas the FEP group does not. (Fig. 5g and Supplementary Fig. 4). No significant effect of precision or group was found for the learning rate decay parameter (Fig. 5h and Supplementary Fig. 4). No group differences were found on the signed and unsigned precision-weighting parameters of the winning model in the HCS and ARMS group, possibly because the model did not provide a good fit for the FEP group (Supplementary Table 4). When we correlated participants’ behavioural response data with data simulated using the individual parameters of the winning model for each group, we found that there was no difference in the amount of variance explained between groups (ANOVA: *F*{2,79} = 0.72, *p* = 0.48, *r* values: HCS = 0.40, ARMS = 0.34, FEP = 0.37), suggesting that the modelling procedure was equally successful across groups (see Supplementary Figs. 8–10).

#### Unsigned prediction errors are precision-weighted bilaterally in the SFC

In bilateral SFC (see methods for ROI derivation) there were brain signals that encoded unsigned prediction error (Right: *T* = 7.33, voxels: 150, *p* < 0.001, [24 4 52]; Left: *T* = 6.78, voxels: 149, *p* < 0.001, [−24 6 52], small-volume correction). There was significantly stronger encoding of unsigned prediction errors in the high-precision condition compared with the low-precision condition in both the right and left SFC, demonstrating precision-weighting (Right: *T* = 3.82, voxels: 66, *p* = 0.011, [22 12 52]; Left: *T* = 3.52, voxels: 45, *p* = 0.025, [−21 −2 52]; small-volume corrected), which is consistent with the effect observed in the dopaminergic modulation study.

In a whole-brain analysis, additional regions demonstrated precision weighting of prediction error: bilateral SFC, right lateral frontal cortex, and medial parietal lobe (Supplementary Table 5).

#### Signed prediction errors

We also tested for signed prediction errors in the ventral striatum and the midbrain using ROI’s based on ref. [16]. However, no significant voxels were found that coded a main effect of signed prediction errors, a precision-weighting effect or a precision by group interaction (all *p* > 0.1).

#### FEP is associated with diminished precision-weighting of the unsigned prediction error signal in the right SFC

**Fig. 6:**
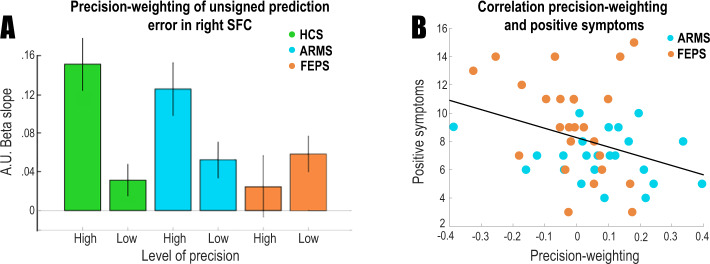
Brain imaging results for the psychosis study. **a** Precision weighting of prediction error in superior frontal cortex region of interest. Precision weighting is significantly diminished in first-episode psychosis. The *y*-axis provides beta estimates for the unsigned prediction error in different precision conditions in arbitrary units (a.u.). **b** Diminished precision-weighting of prediction error is correlated with positive symptoms. Error bars represent standard error of the mean.

There was a significant difference in precision weighting of the unsigned prediction error signal between the FEP group and the control group in the right SFC (*T* = 3.38, voxels: 9, *p* = 0.035, [24 9 48]; small-volume corrected) (see Fig. 6a and Supplementary Fig. 11). Importantly, group differences were not driven by medication as this precision weighting in medicated psychosis patients was not significantly different from patients who non-medicated (*T*{18} = 0.14, *p* = 0.89; analysis conducted on voxels that showed a FEP vs. control group difference), and there was no correlation between medication dose and precision weighting (*r* = 0.17, *p* = 0.52). No voxels differentiated the groups on whole-brain analysis, or left SFC ROI analysis, corrected for multiple comparisons. We tested whether precision weighting in these nine voxels (that differentiated the FEP and control groups) correlated to positive symptom severity (sum of PANSS items P1, 2 and 3). To increase the number of participants for this analysis with a wide variety of symptoms we included both the ARMS group and the FEP group (Fig. 6b). Reduced precision-weighting related to greater positive symptoms (*r* = −0.33, *p* = 0.032), but not when controlling for group (*p* = 0.3). As group and symptoms are confounded given our FEP inclusion criterion of having current delusions and/or hallucinations, and as low sample size limits our statistical power for correlations within group, we also ran an additional analysis including an extra six participants with FEP who did not present with sufficient levels of positive psychotic symptoms to be included in main study (pooled ARMS and FEP, controlling for group *r* = −0.28, *p* = 0.054; see Supplementary Fig. 12).

#### Higher schizotypy is related to decreased performance and diminished precision-weighting of cortical prediction errors in a separate healthy sample

**Fig. 7:**
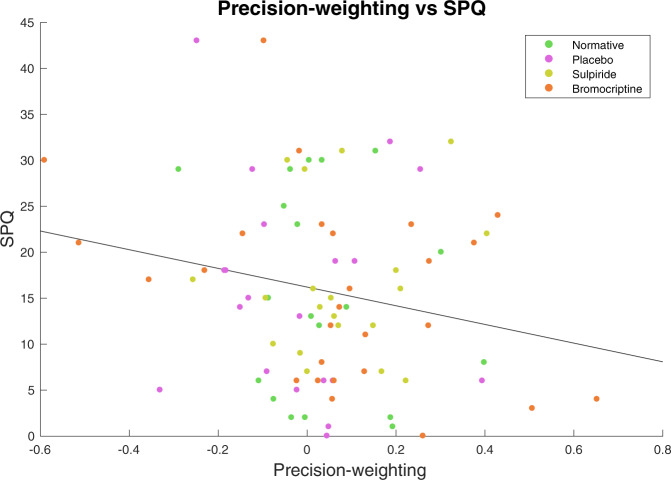
Cortical learning signals and schizotypy. Higher SPQ scores (schizotypy) were correlated with less precision-weighting of the unsigned prediction error signal in the right superior frontal cortex, including when controlling for experimental group; Rho = −0.25, *p* = 0.024.

We next examined the relationship between schizotypy and precision weighting of prediction error in health, free from the possible confounds of medication or illness duration driving effects (Supplementary methods). We pooled the participants in the dopaminergic modulation study (which is the study described in this paper, *N* = 59) and the participants of a previously collected healthy sample (who are from a previously reported study [15], *N* = 27) and tested for a relationship between schizotypy and precision weighting of prediction error, while controlling for experimental group. There was a significant correlation between performance and schizotypy (Rho = −0.23, *p* = 0.034). This was mirrored by a significant brain signal-schizotypy correlation between schizotypy and the extracted right SFC precision-weighting parameter estimates (Rho = −0.25, *p* = 0.024) (Fig. 7). Higher schizotypal personality scores were associated with less cortical precision-weighting of unsigned prediction error signals. No relationship was observed between schizotypy and the main effect of unsigned prediction error (*p* > 0.3), suggesting that the effect is specific to the precision weighting of the unsigned prediction error signal.

## Discussion

In this study we aimed to investigate if precision weighting of signed and unsigned prediction error signals is altered in psychosis. We also tested whether unsigned prediction errors are coded relative to their precision, and whether dopamine modulated this precision weighting in healthy individuals, as this has previously been largely unclear. We found that unsigned prediction errors are coded in the SFC, where the unsigned prediction error signal is coded relative to the precision of environmental outcomes; that the degree of precision-weighting benefits learning, is mediated by dopamine, is perturbed in FEP, and relates to schizotypy in a health. In contrast to previous work, we did not observe significant coding of signed prediction errors relative to their precision, or signed prediction error coding per se, in the healthy controls (or patients) in the psychosis study.

Recent theories [3, 4, 6, 9, 28] have hypothesised that precision weighting of cortical unsigned prediction errors is mediated by neuromodulators (including dopamine), and link a malfunctioning dopamine system to psychosis through aberrant precision-weighting of these prediction errors. However, to our knowledge, no direct evidence for any of these claims exists. Here we showed that separately estimated precision-weighted signed and unsigned prediction errors provided the best description of the behavioural data, thus suggesting that both precision-weighted and unsigned prediction errors should be represented in the brain. The representation of precision-weighted signed prediction errors in subcortical areas was confirmed previously by fMRI in humans [15]. In the present study, we tested the prediction that unsigned prediction errors would be represented in the brain, and found evidence for a precision-weighted cortical representation of unsigned prediction in the bilateral SFC and dorsal anterior cingulate cortex/pre-supplementary motor area (that we collectively referred to as SFC). Cortical precision-weighting was significantly diminished in the sulpiride (dopamine D2 receptor antagonism) group in comparison with the other groups in the right SFC; there was marginal evidence of a medication effect in the dACC. This finding suggests that dopamine plays a key role in the mechanisms underlying precision weighting of unsigned prediction errors. We furthermore found that a greater degree of superior frontal precision-weighting of unsigned prediction error was significantly correlated to performance on the task, where an increase in precision weighting resulted in more accurate predictions of upcoming rewards. These results confirm the prediction that there exist cortical unsigned prediction error signals, which influence performance and are precision-weighted by dopamine.

The coding of unsigned prediction errors in the superior and middle frontal gyri and dACC is in line with earlier findings by Hayden et al. [32] who found unsigned prediction errors in the dACC of monkeys, and with prior fMRI studies in humans [33–38]. Our findings are consistent with those of Katthagen et al. [39], who used reaction time data (rather than choice data) from a human fMRI reversal learning study to derive a relevance-weighted unsigned prediction error signal, which was also represented in the dACC. Our data in the dopaminergic modulation study, replicated in the psychosis study, show (for the first time to our knowledge) that cortical prediction error signals based on choice data are precision-weighted in humans. We note that dopaminergic innervation of cortex is greatest in superior frontal regions [40–42], compatible with the hypothesis that precision-weighting is influenced here by dopaminergic input.

If the precision weighting of prediction errors is important in learning, we can expect aberrant learning to occur when prediction errors are not scaled optimally to the environmental statistics determining the precision of available information. We tested whether this mechanism could be of importance to psychosis, which is characterised by delusional beliefs and hallucinatory perception [9]. Previous work showed aberrant cortical and subcortical prediction error coding in people with psychosis [10, 11, 43]. As psychosis has consistently been associated with dopamine dysfunction [44], it is possible that a dopamine-mediated precision-weighting process would be impaired in psychosis. Indeed, it has been suggested that dopamine dysregulation causes psychosis due to affecting the brain’s capacity to precision-weight prediction error [3]. That is, if unreliable prediction errors were given excessive weight, they could have an exaggerated influence on driving changes in the brain’s model of the world, thereby contributing to the formation of abnormal beliefs. We found several lines of evidence suggesting that FEP in particular is associated with a failure to precision-weight prediction errors. First, FEP was associated with decreased performance on the task. Furthermore, the FEP group did not benefit as much from more precision reward information than the healthy controls and ARMS group did, and computational modelling indicated that the FEP group does not precision-weight prediction errors, as they follow a simple RW learning rule without precision-weighted prediction errors. By contrast, controls and ARMS follow a PH learning rule with precision-weighted prediction errors. This invites the question whether the poor performance of the FEP group might have more to do with the failure to diminish their learning rate appropriately over time (as this is what characterises a PH model). However, subsequent analysis revealed that whereas controls and ARMS show a clear effect of precision on learning rate, the FEP group does not. In contrast, no differences were found for the decay parameter, suggesting that the differences lie in how much prediction errors are used in different precision conditions. Third, neural evidence suggests that the FEP group does not precision-weight cortical prediction errors to the extent that healthy controls do, and that the degree of neural abnormality may relate to positive psychotic symptom severity (we acknowledge that the modest patient sample size and marginal significance of the correlation is not conclusive, though the relation is supported by the finding that in healthy individuals the degree of cortical precision-weighting relates to schizotypy, consistent with a continuum model of psychosis).

Several other studies have used this computational framework to study learning in individuals with psychosis that imply a failure to precision-weight prediction errors. Ref. [45] used hierarchical Bayesian models to make inferences about the way individuals with psychosis respectively form beliefs about the environment. Critically in these models a prediction error is weighted by the precision of beliefs regarding cue-outcome contingencies, and the volatility of these relationships [2]. As such, these models imply precision weighting of prediction, however they do not test the degree to which these prediction errors are precision-weighted explicitly. Our results complement these studies and provide an additional direct test of the degree of precision-weighting of prediction errors in psychosis.

A previous study has reported differences between healthy controls and individuals with schizophrenia in the degree to which they adapt the coding of value to the variability in the environment [46]. This process of adaptive coding is similar to precision weighting of unsigned prediction errors, as it reflects the brain’s capacity to scale neural signals to what is referred to as economic ‘risk’, in other words the spread of possible reward outcomes. In combination with the present findings, psychotic disorder might be associated with a broader failure to adapt neural signals to the statistics of the environment.

We thus conclude that there is evidence for a diminishment in precision weighting of unsigned prediction errors in individuals with FEP. This was most strongly related to the intensity of the positive symptoms experienced by the patients in this study. Our current study provides evidence for a key hypothesis in the field of predictive coding theories of psychosis, which is that psychosis is associated with a failure to accurately take into account the reliability of new information, leading to the formation of aberrant inferences about the world, predisposing to delusional beliefs. The finding that the degree of precision weighting of cortical prediction errors is modulated by dopamine, combined with the finding of abnormal precision-weighting in psychosis, is consistent with the posit that the origins of the precision-weighting deficit in psychosis are dopaminergic. However, it is challenging to theoretically accommodate our cortical dopaminergic results into a model of psychosis given the very well established prior findings linking striatal (rather than cortical) alterations in dopamine transmission to psychotic symptoms [47]. We note that although we demonstrate dopaminergic modulation of the degree of cortical precision-weighting in healthy volunteers, there may be other neurotransmitters that also contribute to this process. As we did not measure dopamine function in the clinical studies, it remains possible that the patient cortical and behavioural deficits are secondary to non-dopaminergic mechanisms. Pharmacological fMRI in patients, and combined fMRI and PET studies (including dopaminergic ligands) in patients, will be important in future work aiming to reconcile predictive coding models of psychosis with the dopamine hypothesis of psychosis.

A significant limitation of this study is that we did not replicate our previous finding of significant precision-weighting of signed prediction errors in the midbrain and ventral striatum in the controls (or indeed patients) of the psychosis study [15, 16]; this meant we could not examine whether or not the precision of signed prediction error signals differs in patients and controls. As data for the psychosis study were collected using a different MRI scanner, head coil and imaging sequence than the dopamine modulation study [16], it is possible that the signal to noise ratio in the psychosis study was lower in subcortical and midbrain areas. In addition, there were slight differences in the experimental design between the two studies (please see ‘Methods’), which might have rendered the design in the psychosis study less sensitive to detect precision weighting of signed prediction errors. It is important to test the replicability of our previous findings in future work.

In conclusion, we found evidence of precision-weighted unsigned prediction errors in the superior frontal and dorsal anterior cingulate cortices. Furthermore, we found that the precision weighting of prediction errors was modulated by the dopaminergic antagonist sulpiride, and we found that the degree of precision weighting in this area was correlated to performance on the task, providing evidence for the first time that dopamine plays a role in precision weighting of unsigned prediction error brain signals during learning. Healthy people, but not patients with FEP, take into account the precision of the environment and unsigned prediction errors when updating beliefs; accordingly, the cortical unsigned prediction error signal is abnormal in psychotic illness, and relates to trait levels of schizotypy in the healthy population, implicating it as a key mechanism underlying the pathogenesis of psychotic symptoms.

## Supplementary information

Supplement

## Data Availability

For data requests please contact the authors or, for the clinical study, openNSPN@medschl.cam.ac.uk.

## Code availability

Code for computational modelling is available at https://github.com/kelly-diederen/prediction-error.
