# Correction: Precision weighting of cortical unsigned prediction error signals benefits learning, is mediated by dopamine, and is impaired in psychosis

**DOI:** 10.1038/s41380-020-00919-9

**Published:** 2020-10-14

**Authors:** J. Haarsma, P. C. Fletcher, J. D. Griffin, H. J. Taverne, H. Ziauddeen, T. J. Spencer, C. Miller, T. Katthagen, I. Goodyer, K. M. J. Diederen, G. K. Murray

**Affiliations:** 1 Department of Psychiatry, University of Cambridge, Cambridge, UK; 2 Wellcome Trust MRC Institute of Metabolic Science, Cambridge Biomedical Campus, Cambridge, UK; 3 Cambridgeshire and Peterborough NHS Trust, Cambridge, UK; 4 Department of Psychosis studies, Institute of Psychiatry, Psychology and Neuroscience, King’s College London, London, UK; 5 Department of Psychiatry and Psychotherapy, Charité—Universitätsmedizin Berlin, Corporate member of Freie Universität Berlin, Humboldt-Universität zu Berlin, Berlin Institute of Health, Berlin, Germany

**Keywords:** Neuroscience, Schizophrenia, Psychology

Correction to: *Molecular Psychiatry*

10.1038/s41380-020-0803-8

published online 24 June 2020

Following publication of this article, the authors noticed an error in Fig. 6. In the original figure, the calculation of the positive symptoms score for the graph in panel B was different to the calculation described in the text, and the scale bar on the *y*-axis of the graph in panel B was incorrect. Additionally, the colours on the data points in Fig. 6B were unclear due to a production error. Figure 6 has now been replaced with a corrected version. The original, incorrect version of Fig. 6 is displayed below for reference.

**Fig. 6:**
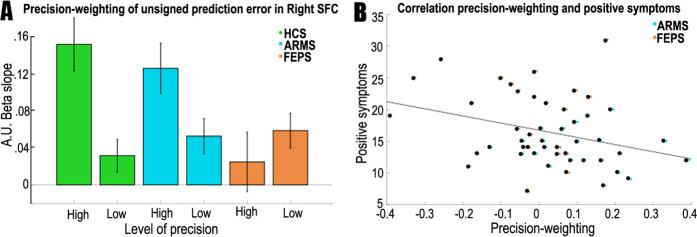
.

